# Phenotypic plasticity and genetic diversity shed light on endemism of rare *Boechera perstellata* and its potential vulnerability to climate warming

**DOI:** 10.1002/ece3.10540

**Published:** 2023-09-15

**Authors:** Jennifer Nagel Boyd, Carol Baskauf, Annie Lindsay, Jill T. Anderson, Jessica Brzyski, Jennifer Cruse‐Sanders

**Affiliations:** ^1^ Department of Biology, Geology, and Environmental Science University of Tennessee at Chattanooga Chattanooga Tennessee USA; ^2^ Department of Biology Austin Peay State University Clarksville Tennessee USA; ^3^ Department of Genetics, Odum School of Ecology, Davison Life Sciences University of Georgia Athens Georgia USA; ^4^ Department of Biology Seton Hill University Greensburg Pennsylvania USA; ^5^ State Botanical Garden of Georgia University of Georgia Athens Georgia USA

**Keywords:** acclimation, adaptation, *Boechera laevigata* (smooth rockcress), *Boechera perstellata* (Braun's rockcress), Brassicaceae, conservation, genetic diversity, plasticity, rare species

## Abstract

The rapid pace of contemporary environmental change puts many species at risk, especially rare species constrained by limited capacity to adapt or migrate due to low genetic diversity and/or fitness. But the ability to acclimate can provide another way to persist through change. We compared the capacity of rare *Boechera perstellata* (Braun's rockcress) and widespread *B. laevigata* to acclimate to change. We investigated the phenotypic plasticity of growth, biomass allocation, and leaf morphology of individuals of *B. perstellata* and *B. laevigata* propagated from seed collected from several populations throughout their ranges in a growth chamber experiment to assess their capacity to acclimate. Concurrently, we assessed the genetic diversity of sampled populations using 17 microsatellite loci to assess evolutionary potential. Plasticity was limited in both rare *B. perstellata* and widespread *B. laevigata*, but differences in the plasticity of root traits between species suggest that *B. perstellata* may have less capacity to acclimate to change. In contrast to its widespread congener, *B. perstellata* exhibited no plasticity in response to temperature and weaker plastic responses to water availability. As expected, *B. perstellata* also had lower levels of observed heterozygosity than *B. laevigata* at the species level, but population‐level trends in diversity measures were inconsistent due to high heterogeneity among *B. laevigata* populations. Overall, the ability of phenotypic plasticity to broadly explain the rarity of *B. perstellata* versus commonness of *B. laevigata* is limited. However, some contextual aspects of our plasticity findings compared with its relatively low genetic variability may shed light on the narrow range and habitat associations of *B. perstellata* and suggest its vulnerability to climate warming due to acclimatory and evolutionary constraints.

## Graphical abstract

The question of why some species are rare while others are common is an enduring one with implications for ecological theory and the conservation of biodiversity. Our research reveals some limited differences in plasticity between rare *B. perstellata* and widespread *B. laevigata* that are dependent on various abiotic factors. These differences could help to explain the narrow range and habitat specificity of *B. perestellata*, while highlighting its potential vulnerability to future environmental change.

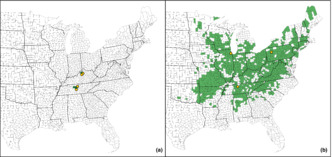

## 1 INTRODUCTION

Biodiversity is at risk worldwide due to dramatic rates of environmental change, often as a result of anthropogenic activities (Knapp et al., 2021; Malhi et al., 2020). Rare species are especially susceptible to extinction in the face of such change (Mouillot et al., 2013), and as such, rare species often drive declines in the biodiversity of communities and systems (Dee et al., 2019). Species can persist through environmental change via migration to more suitable habitat (Chen et al., 2011; Crickenberger & Wetheym, 2018; Hickling et al., 2006; Parmesan, 2006), adaptation to changed conditions in existing habitat (Hamann et al., 2020; Jump & Peñuelas, 2005; Sheth et al., 2018), and/or acclimation to changed conditions (Chevin et al., 2010; Nicotra et al., 2010; Seebacher et al., 2015). But relative to more common species, the ability of rare species to migrate and/or adapt could be impeded by their low fitness (Boyd, Anderson, et al., 2022; Iverson et al., 2004) and/or genetic diversity (Cole, 2003; Gitzendanner & Soltis, 2000; Leimu & Fischer, 2008). Given these constraints, the ability to acclimate to relatively rapid environmental change through phenotypic plasticity could be an important pathway to persistence for rare species.

Relative to other taxonomic groups, plants are generally characterized by high levels of plasticity (Sultan, 2000). But plant species, populations, and individuals can exhibit dramatic differences in plasticity that may influence their responses to environmental change in different ways (Balaguer et al., 2001; Cleavitt, 2002; Dangremond et al., 2015; Godoy et al., 2012; Nicotra & Davidson, 2010; Osunkoya & Swanborough, 2001; Pohlman et al., 2005; Stamp & Hatfield, 2020; Sultan, 2000; Valladares et al., 2000, 2007). Plasticity has been underexplored in the context of the causes and consequences of plant species rarity (Boyd, Anderson, et al., 2022; but see Boyd, Odell, et al., 2022; Hirst et al., 2017; Liao et al., 2006; Lovell & McKay, 2015; Rutherford et al., 2017), although it has been long suggested that rare species could be constrained by low plasticity relative to more common species (Murray et al., 2002). Such constraints could explain both the narrow ranges and habitat specificity of many rare species, as well as their declines in the face of environmental change (Nicotra et al., 2010). Studies that compare the plasticity of rare and common species in the context of environmental change could test the hypothesis that geographically restricted species and/or habitat specialists may be limited by an inability to acclimate to broader environmental conditions (Boyd, Anderson, et al., 2022) and provide guidance for rare species conservation during a time of rapid environmental change (Bevill & Louda, 1999). Congeneric comparisons could be especially useful in advancing our understanding of the causes and consequences of species rarity by controlling for differences in life history and phylogeny (Combs et al., 2013; Farnsworth, 2006; Godt & Hamrick, 2001; Kunin & Gaston, 1997; Murray et al., 2002).

**FIGURE 1:**
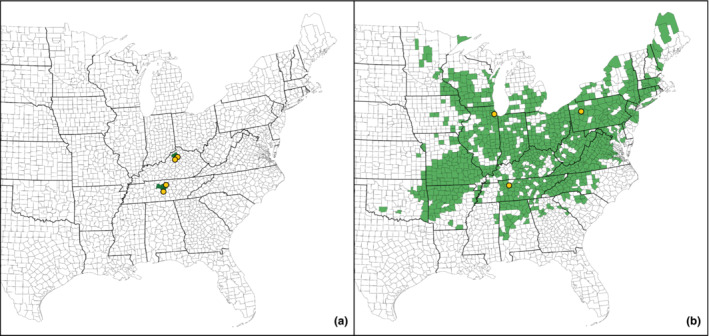
Maps of the distribution of rare *Boechera perstellata* (a) and widespread *B. laevigata* (b) in the eastern United States at the county level (green shaded areas) and locations of the natural populations of each species from which seeds were collected (yellow dots). While *B. perstellata* only occurs in the United States, the range of *B. laevigata* extends into southeastern Canada.

*Boechera perstellata* (E. L. Braun) Al‐Shehbaz (Braun's rockcress) is a rare endemic plant species with a disjunct distribution consisting of a small number of populations along wooded limestone outcrops in central Tennessee and north‐central Kentucky, USA (USFWS, 1995, 1997, 2004) separated by a ~250‐km gap (Figure 1a). Previous research revealed that *B. perstellata* has very low levels of genetic diversity (Baskauf et al., 2014) but that study did not include relative comparisons with more common species. In contrast, widespread *B. laevigata* (Muhl. Ex Willd.) Al‐Shehbaz (smooth rockcress) occurs throughout much of the eastern United States (Figure 1b) as well as southern Quebec in habitat that is largely similar to that of *B. perstellata* (Al‐Shehbaz & Windham, 2010; Bloom et al., 2002) but across a broader range of substrates (Kiefer et al., 2009). Populations of *B. laevigata* also have been reported along railroad tracks suggesting that its distribution in some locations may have been influenced by human activities (Kiefer et al., 2009).

To investigate the role of acclimation as a cause and/or consequence of species rarity, we compared the phenotypic plasticity of rare *B. perstellata* and widespread *B. laevigata* in response to a suite of abiotic factors—light, temperature, and water availability. We also compared the evolutionary potential of these species through investigations of their population‐level genetic diversity. Given its narrow geographic distribution and less diverse habitat associations than *B. laevigata*, we hypothesized that rare *B. perstellata* would have lower plasticity and genetic diversity than its more common congener. In particular, we expected *B. perstellata* to have comparably lower plasticity in biomass allocation traits associated with light and water acquisition in the context of the availability of these resources. In response to warming, we expected *B. perstellata* to exhibit lower plasticity in traits associated with size and leaf morphology, which have been found to be particularly responsive to temperature (see Stotz et al., 2021). We also expected that populations of *B. perstellata* would exhibit less intraspecific variation in plasticity and within‐population genetic diversity—as causal and/or consequential to rarity—than would populations of *B. laevigata*, although we expected some significant divergence between the disjunct Tennessee and Kentucky populations of *B. perstellata*.

## 2 MATERIALS AND METHODS

### 2.1 Study system

*Boechera perstellata* is a rare species restricted to just three counties each in the Central Basin region of Tennessee, USA and the Bluegrass region of north‐central Kentucky, USA (USFWS, 2004, 2018). This rare species is associated with almost dry to moderately moist, shady, steep slopes on limestone outcrops, often in sheltered areas with limited competition from surrounding vegetation (USFWS, 2004, 2018). At present, there are 47 occurrences of *B. perstellata* known from throughout its range with the vast majority occurring in Kentucky (USFWS, 2018). While most occurrences consist of few individuals, some are comprised of >1000 individuals (NatureServe, 2005). Given its small geographic range and narrow habitat specificity (i.e., limestone outcrops) but large size of some populations, we categorize *B. perstellata* as “endemic” (see Rabinowitz, 1981), which is the most common type of species rarity (May, 1988; Rabinowitz, 1981). Given its endemism and associated conservation concerns, *B. perstellata* is listed as federally endangered (USFWS, 2018). Like its rare congener, widespread *B. laevigata* often occurs on limestone rock outcrops, but this species also can be found growing on open rocky or gravel areas throughout a wider range that encompasses much of eastern North America (Al‐Shehbaz, 1988).

Despite their phylogenetic relatedness, comparisons of *Boechera* species can be complicated by differences in their life histories and reproductive and ploidy patterns. While the genetic diversity of *B. perstellata* has been compared with that of short‐lived perennials in particular (Baskauf et al., 2014), its lifespan is not well known beyond the persistence of woody old growth for several years (Braun, 1956). Depending on plant size and resource availability, *B. laevigata* has been described as being able to function as a short‐lived perennial or biennial (Bloom et al., 2002, 2003). While most *Boechera* species are sexual diploids, some species are polyploid, and some reproduce asexually through seed (apomixis; Dobeš et al., 2006). Both *B. perstellata* and *B. laevigata* are typically diploid, sexually reproducing species, and *B. laevigata* is known to be capable of reproducing by both outcrossing and selfing (Bloom, 1988). Recent evidence also suggests that a few populations of this species are reproducing by apomixis (Carman et al., 2019; M. Windham, personal communication). The model organism *Arabidopsis thaliana* is in the same family (Brassicaceae) as *Boechera* species, and the *Boechera* genus has become a model system for testing ecological and evolutionary questions given its relatively small genomes and occurrence in natural habitats throughout North America (Rushworth et al., 2011; Schranz et al., 2007).

### 2.2 Seed collection and propagation

Several populations were sampled from throughout the range of each *Boechera* species to represent a range of genetic and phenotypic variation within each species. Seeds of *B. perstellata* were collected from one population each in Rutherford County and Smith County, Tennessee, USA (referred to as TN1 and TN2, respectively) and two populations from Franklin County, Kentucky, USA (KY1, KY2; Figure 1a; Table S1 in Supporting Information for this article). The sites for collection of *B. perstellata* seed were determined in cooperation with the USFWS given the federally protected status of this species. Sampled populations in Tennessee were separated by ~50 km while sampled populations in Kentucky were separated by ~12 km such that gene flow between populations would have been unlikely. Seeds from parent individuals of *B. laevigata* were collected from three populations: Cheatham County, Tennessee, USA (TN); Cook County, Illinois, USA (IL); Clarion County, Pennsylvania, USA (PA; Figure 1b; Table S1). All *B. perstellata* seeds and *B. laevigata* seeds from the Tennessee population were collected by the authors, laboratory personnel, and/or professional contacts. Vouchers for the *B. laevigata* populations were deposited in the Austin Peay State University Herbarium (ASPC). From each population of each species, we collected numerous seeds from each of 12–16 distinct parent individuals in separate paper bags to retain maternal information. All seed was collected from the field in spring/summer 2018. All collected seeds were stratified for 4 months prior to planting.

Following stratification, we sowed 6–8 seeds representing half to full siblings per maternal parent from each population into multiple 7‐cm^2^, 8.5‐cm‐deep (~0.4 L) pots filled with a commercial potting medium (Pro‐Mix Bx Biofungicide + Mycorrhizae; Premier Tech Horticulture). Although this growth environment differs from the limestone outcrops with which *B. perstellata* is generally associated, our use of potting mix was informed by our previously successful protocol for growing rare species associated with rock substrates and with consideration of our limited seed supply given the endangered status of this species (see Boyd, Odell, et al., 2022). Using a rich and common potting medium for both *B. perstellata* and *B. laevigata* also allowed us to control for the influence of edaphic factors on measured outcomes. Four pots from each parent individual were randomly assigned such that one pot was housed in each of four growth chambers (PGR 15, Conviron Controlled Environments Limited, Winnipeg, Manitoba, Canada). During a 1‐month germination period, all chambers were to set to provide a 12‐h photoperiod at constant 25°C, and we watered pots as needed to maintain moist soil. Following the germination period, we thinned each pot so that it included the single individual that exhibited the earliest third leaf development. These individuals were then transplanted into separate 11‐cm^2^, 9.5‐cm‐deep (~1.1 L) pots containing the same potting medium to minimize the potential for plants to become root‐bound for the duration of the project (*n* values reported in Table S2).

### 2.3 Environmental treatments

Following transplantation, we programmed the four growth chambers to allow us to assess plasticity of *B. perstellata* and *B. laevigata* in response to light, temperature, and water as modified from methods previously described by Boyd, Odell, et al. (2022). We generally aimed to impose contrasting levels of these abiotic factors across which plasticity could be tested that were also informed by the general direction and specifics of likely environmental change when possible, although this determination was more apparent for some factors than others. We programmed an “ambient” chamber to be used in all of the plasticity assessments to provide temperature of 20–30°C (nighttime–daytime) based on regional weather records and a 12‐h photoperiod with a maximum daily light level of 250 μmol photons/m^2^/s in accordance with our field measurements of photosynthetically active radiation (PAR) in *B. perstellata* habitat during the growing season. The availability of water in *B. perstellata* habitat is a complex abiotic factor to assess due to the potential for small‐scale temporal variation associated with precipitation events. But given the generally well‐drained substrate associated with *B. perstellata*, we imposed a level of water stress as a baseline in our plasticity experiments by watering pots in the ambient chamber to 50% field capacity every 2 days as determined by weighing a subset of pots of each species in accordance with previously described methods (see Boyd, Odell, et al., 2022). The other three growth chambers were programmed to provide the same conditions as the “ambient” chamber but each with a contrasting level of a single condition (light, temperature, or water) across which plasticity could be assessed. Specifically, in one chamber, we doubled light availability to a maximum daily level of 500 photons μmol/m^2^/s to represent a dramatic increase in light, as could be associated with timber harvesting and/or other land‐use change associated with deforestation. In another chamber, we simulated average projections of global temperature increase for this century (Pörtner et al., 2022) by exposing plants to 22–32°C (nighttime–daytime). In the final chamber, we doubled water availability by watering plants to 100% field capacity every 2 days. Projections suggest that precipitation amounts and heavy precipitation events will increase in eastern North America (IPCC, 2023); however, we concede that the effects of such change on water availability will be complicated by edaphic factors and effects on evapotranspiration. As such, while allowing us to test for plasticity, the “real world” translation of our water treatment levels is limited. We grew all individuals in the growth chambers for 4 months following treatment initiation during which time growth data were collected. To minimize any effects of chamber and pseudoreplication (Gibson, 2014), we reassigned treatment levels to each chamber monthly and moved all plants accordingly. Within chambers, we rotated the positions of pots each week to control for spatial differences in environmental conditions.

### 2.4 Growth, allocation, and leaf morphology measures

We recorded plant height and counted the numbers of leaves of each individual at 4 months after treatment initiation. We then harvested all individuals to yield measures of growth and biomass allocation. We quantified root length (cm) as the distance from the tip of the longest root to the beginning of the green shoot when freshly harvested plants were held upright. The single youngest fully expanded leaf from each individual was removed, scanned to determine its area, and dried in a laboratory oven to calculate specific leaf area (SLA; cm^2^/g) for each individual. Concurrently, we separated the remains of each harvested plant into shoots and roots, which were also dried to determine shoot dry mass (g), root dry mass (g), and total dry mass (g). We included the dry mass of leaf material removed for SLA measurements and genetic investigations (described in later subsections) in our determinations of shoot dry mass. We calculate mass‐based root‐to‐shoot ratio (RSR_mass_; g/g) by dividing root dry mass by shoot dry mass, length‐based root‐to‐shoot ratio (RSR_length_; cm/cm^2^) by dividing root length by shoot length, and specific root length (SRL; cm/g) by dividing root length by root dry mass.

### 2.5 Analyses of survival and trait plasticity

We analyzed the probability of survival to harvest as a function of species, growth chamber treatment, and their interaction in a generalized linear mixed model framework with a binomial distribution and a random effect for source population in the glmer function of the lme4 R package.

To analyze plasticity of growth, allocation, and leaf morphological traits, we fit linear mixed‐effects models using the lmer function of the R package lme4 (Bates et al., 2015) with species, abiotic treatment (i.e., light, temperature, and water), and their interaction as explanatory factors. We included population as a random effect in these models. A significant interaction indicated that *B. perstellata* and *B. laevigata* responded differently to a change in the environmental factor (i.e., that the two species exhibited differences in plasticity; see Boyd, Odell, et al., 2022). To account for the numerous traits analyzed to assess plasticity, we used corrected *p‐*values to minimize the false discovery rate (FDR; Benjamini & Hochberg, 1995). Results of statistical tests were considered significant if FDR‐corrected *p* ≤ .05. When there was a significant species × abiotic treatment interaction, we contrasted estimated means of each species between abiotic treatment levels with the R package emmeans (ver. 1.8.7).

We used a relative distance plasticity index (RDPI; see Valladares et al., 2006) to calculate plasticity of traits in the context of our light, temperature, and water treatments as previously detailed in these contexts by Boyd, Odell, et al. (2022). The RDPI, which can range from 0 (no plasticity) to 1 (maximum plasticity), is based on the absolute phenotypic distances of 1.8.7 genotypes across different environments and allows for statistical comparison of plasticity for species and populations (Valladares et al., 2006). To assess the potential influence of plasticity on fitness, we conducted across‐environment multivariate genotypic selection analysis (Stinchcombe et al., 2004; Van Kleunen & Fischer, 2001) with the lmer (linear mixed model) function of the R package *lme4* (ver. 1.1‐21; Bates et al., 2015). We focused these analyses on traits with significant effects of abiotic factor or abiotic factor × species interactions (i.e., traits for which we found significant evidence for plasticity). We analyzed fitness as a function of mean trait values, RDPI, species, and two‐way interactions between mean trait values and species and RDPI and species in separate models for each manipulated environmental condition (i.e., temperature and water). The average total dry biomass calculated across ambient and manipulated environmental conditions (e.g., ambient and increased temperature) for each maternal line (i.e., mean total biomass) served as the fitness proxy in these regressions because total biomass was measurable for all individuals included in our experiment and is generally associated positively with reproductive output (Weiner et al., 2009). A significant effect of RDPI in a trait on fitness suggests selection for plasticity in both species if the slope is positive and selection against plasticity if the slope is negative. A significant interaction between species and RDPI suggests that the magnitude or direction of selection differs between species. Because we conducted two separate analyses of selection on plasticity, we used a Bonferroni‐corrected *α* = 0.025 (= 0.05/2) to assess significance and control for type I errors. We used the predictorEffects function of the R package *effects* (ver. 4.2‐0; Fox & Weisberg, 2018) to visualize selection landscapes from these multiple regression models as partial residual plots.

### 2.6 DNA extraction and microsatellite genotyping

Leaf tissue was collected from all *B. perstellata* and *B. laevigata* individuals included in the growth chamber experiments prior to harvest. Collected leaf tissue was dried on silica gel and ground in a bead mill (MM301; Retsch). DNA was extracted from leaf material with the E.Z.N.A. SP Plant DNA Kit (Omega Bio‐Tek, Inc.). Previously designed microsatellite primers were initially evaluated using touchdown polymerase chain reaction (PCR) analysis (see Baskauf et al., 2014), except for ICE4, F03, and H06, for which constant annealing temperatures of 50, 50, and 55°C, respectively, were used. Fluorescent labeling protocols followed Schuelke (2000) as modified by Baskauf et al. (2014), except that six primers (F03, G03, G06, G08, G09, and H06) had a short tag (GTTTCTT) attached to the 5′ end of the reverse primer. PCR products were multiplexed, and fragment analysis was completed with an ABI 3130XL DNA Analyzer (Applied Biosystems) by the University of Tennessee Health Science Center (Memphis, Tennessee, USA). Individuals were genotyped using GeneMarker v1.97 (SoftGenetics, LLC). Alleles were manually identified and verified by two people.

### 2.7 Analysis of genetic diversity

In total, 185 *B. perstellata* and 151 *B. laevigata* individuals were included in most analyses. We used GenAlEx v6.503 (Peakall & Smouse, 2006, 2012) to calculate standard genetic diversity statistics, including percentage of polymorphic loci (*P*), alleles per locus (*A*), observed (*H*_o_), and Nei's (1978) unbiased expected heterozygosity (*H*_e_). Examination of alleles indicated that the Illinois population (IL) of *B. laevigata* is likely apomictic and that the Pennsylvania population (PA) of this species experiences high levels of inbreeding. To account for possible apomixis, we used the R package Poppr v4.0.3 (Kamvar et al., 2014), generating a unique multilocus genotype dataset with a clonal threshold of 0 for an analysis of clonal diversity. In addition to the number of unique multilocus genotypes (MLG), clonal diversity measures included the expected number of multilocus genotypes (eMLG), the Shannon–Wiener index of MLG diversity using natural logarithm (*H*; Shannon, 1948), and Simpson's (1949) index (*D*). To assess population structure and grouping of individuals in the study, we used a discriminant analysis of principal components (DAPC; Jombart et al., 2010; Machado et al., 2021) with the R package adegenet v2.1.3 (Jombart, 2008). DAPC is a nonparametric multivariate analysis that combines principal component analysis, K‐means clustering, and discriminant analysis and that does not require assumptions of populations being in Hardy–Weinberg equilibrium or the absence of linkage disequilibrium (Alhusain & Hafez, 2018). We used the randomization‐based testing of FSTAT ver. 2.9.4 (Goudet, 1995, 2003) to compare the genetic diversity of *B. perstellata* and *B. laevigata* in terms of allelic richness (*Rs*, a measure of the number of alleles independent of sample size) and sample‐size weighted estimates of observed heterozygosity and expected heterozygosity (*H*_s_, “gene diversity”; Nei, 1987) using 1000 permutations. For the FSTAT comparison of the species, a unique‐MLG‐only dataset was used for the presumed apomictic IL population of *B. laevigata* while full datasets were used for the sexually reproducing populations of both species (as detailed by Lindsay, 2021).

## 3 RESULTS

### 3.1 Survival

Across all treatments, 84.5% of *B. perstellata* individuals and 92% of *B. laevigata* individuals used in our plasticity experiment survived to harvest (Table S2), but the probability of survival varied as a function of treatment. Specifically, individuals across both species had a significantly lower probability of survival with increased watering than in ambient conditions (Figure S1). In contrast, the probability of survival did not differ between species across treatments. The interaction of species and treatment also did not significantly affect survival. No individuals of either species flowered during the experiment.

### 3.2 Species growth, allocation, and leaf morphology

There were significant differences in several traits of the rare and widespread *Boechera* species across levels of abiotic treatments (Table S3). Specifically, in all three comparisons (i.e., ambient vs. increased light, ambient vs. increased temperature, ambient vs. increased water), *B. perstellata* individuals had more leaves and greater SLA than *B. laevigata* individuals (Table S4). In the ambient versus increased water comparison only, *B. laevigata* had greater root mass and RSR_mass_ than *B. perstellata* individuals across treatment levels (Table S4).

### 3.3 Phenotypic plasticity

**FIGURE 2:**
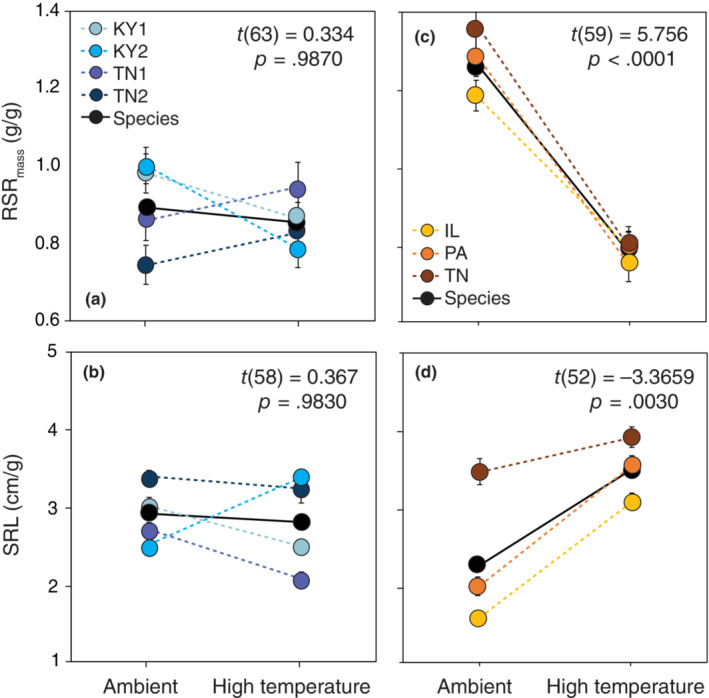
Reaction norms of the means of mass‐based root‐shoot ratio (RSRmass) and specific root length (SRL) of rare *Boechera perstellata* (a, b) and widespread *B. laevigata* (c, d) grown in ambient conditions of *B. perstellata* habitat and with increased temperature. Solid lines and symbols depict species‐level means and norms; dashed lines and colored symbols depict population‐level means and norms. Error bars represent ±1 standard error of the mean; *p*‐values denote the significance of differences in species means between abiotic treatment levels (i.e., species‐level plasticity).

**FIGURE 3:**
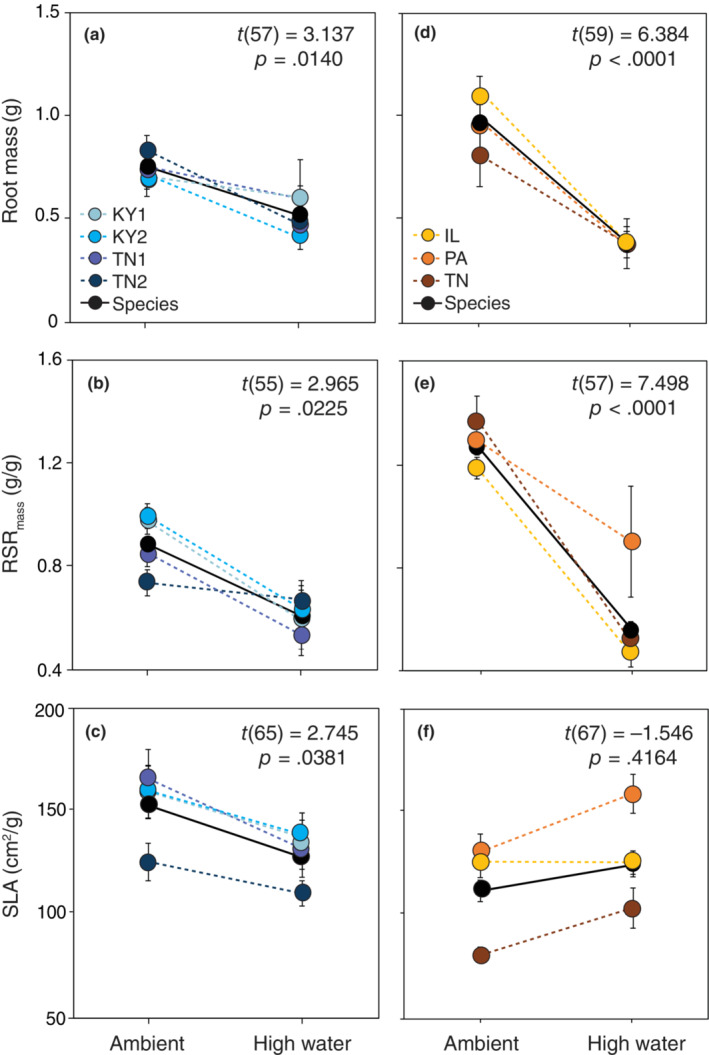
Reaction norms of the means of root mass, mass‐based root‐shoot ratio (RSRmass), and specific leaf area (SLA) of individuals of rare *Boechera perstellata* (a–c) and widespread *B. laevigata* (d– f) grown with water stress and with increased water availability. Solid lines and symbols depict species‐level means and norms; dashed lines and colored symbols depict population‐level means and norms. Error bars represent ±1 standard error of the mean; *p* values denote the significance of differences in species means between abiotic treatment levels (i.e., species‐level plasticity).

We found no evidence for phenotypic plasticity of either *B. perstellata* or *B. laevigata* in response to light availability (see results for main effects of abiotic factor and species × abiotic factor interaction in Table S3). However, there were some differences in the plasticity of root growth and allocation of rare and widespread *Boechera* species in response to both warming and increased water availability (see results for species × abiotic factor interaction in Table S3). In response to warming, widespread *B. laevigata* allocated less of its total biomass to roots (i.e., lower RMR_mass_) but had greater SRL (Figure 2). In contrast, *B. perstellata* did not exhibit plasticity of these or any other any measured traits in the context of temperature change (Figure 2). In response to increased water availability, individuals of both *B. perstellata* and *B. laevigata* produced less root biomass and allocated less of their total biomass to roots (Figure 3). In addition, increased water availability was associated with greater SLA of rare *B. perstellata* but no significant change in the SLA of *B. laevigata* (Figure 3). Selection via biomass did not operate significantly on trait plasticity under temperature manipulation (Table S5), but selection did operate significantly on trait plasticity of RSR_mass_ under water manipulation (Table S6). Specifically, there was selection for plasticity of RSR_mass_ (i.e., plasticity of this trait was adaptive) in both *Boechera* species (Figure S2).

### 3.4 Genetic diversity

A total of 17 microsatellite loci amplified in all populations for both species and were polymorphic in at least one of the species (Table S7). Standard genetic diversity measures for plants from the growth chamber experiments indicated that, on average, rare *B. perstellata* is less variable than widespread *B. laevigata* (Table 1). Across populations, mean alleles per locus and percent polymorphic loci were about twice as high, *H*_e_ was more than four times as high, and *H*_o_ was two orders of magnitude higher for *B. laevigata* than for *B. perstellata*. However, the sampled populations of *B. laevigata* were not homogenous in their genetic diversity, with two populations having relatively high diversity but one population exhibiting almost no diversity. As a result, the FSTAT analysis indicated that only *H*_o_ differed significantly between the rare and widespread *Boechera* species (*p* = .0280), while allelic richness and gene diversity (the sample‐size weighted *H*_e_) did not vary significantly between species (Table 2). At the species level, the three *B. laevigata* populations represented in these experiments revealed a total of 86 alleles across all the sampled loci, while the four populations of *B. perstellata* totaled 40 alleles across the same loci.

**TABLE 1:** Measures of genetic diversity of individuals grown in growth chambers from seed collected from four natural populations of rare *Boechera perstellata* and three populations of widespread *B. laevigata.*

Population or species	*N*	Alleles Per locus	Private alleles	Polymorphic loci	*H*_o_	*H*_e_
KY1	40.0 ± 0.0	1.2 ± 0.1	1	17.7	0.001 ± 0.001	0.055 ± 0.036
KY2	41.0 ± 0.0	1.3 ± 0.2	3	17.7	0.003 ± 0.003	0.052 ± 0.036
TN1	51.9 ± 0.1	1.4 ± 0.2	12	23.5	0.003 ± 0.003	0.087 ± 0.042
TN2	54.9 ± 0.1	1.4 ± 0.1	4	29.4	0.002 ± 0.001	0.084 ± 0.040
*B. perstellata*	47.0 ± 0.8	1.3 ± 0.1	5 ± 2.4	22.1 ± 2.8	0.002 ± 0.000	0.070 ± 0.019
IL	60.0 ± 0.0	2.3 ± 0.2	11	82.4	0.804 ± 0.094	0.430 ± 0.050
PA	38.0 ± 0.0	1.0 ± 0.0	1	0.0	0.000 ± 0.000	0.000 ± 0.000
TN	53.9 ± 0.1	3.6 ± 0.7	46	82.4	0.224 ± 0.034	0.469 ± 0.061
*B. laevigata*	50.6 ± 1.3	2.3 ± 0.3	19.3 ± 13.6	54.9 ± 27.5	0.343 ± 0.058	0.300 ± 0.040

*Note*: Measures include the number of alleles per locus, number of private alleles, percentage of polymorphic loci, direct count of heterozygosity (*H*_o_), and Nei's, 1978 unbiased estimate of mean expected heterozygosity (*H*_e_). Values shown are means across 17 microsatellite loci ± standard errors (when applicable). Populations of *B. perstellata*: Franklin County, Kentucky, USA (KY1, KY2); Rutherford County, Tennessee, USA (TN1); Smith County, Tennessee (TN2). Populations of *B. laevigata*: Cook County, Illinois, USA (IL); Clarion County, Pennsylvania, USA (PA); Cheatham County, Tennessee, USA (TN).

**TABLE 2:** Statistical comparison of several genetic diversity statistics averaged across 17 microsatellite loci for four *Boechera perstellata* and three *Boechera laevigata* populations, using FSTAT.

Statistic	*B. perstellata*	*B. laevigata*	*p*‐Value
Allelic richness (*R*_*S*_)	1.223	2.071	.1430
Observed heterozygosity (*H*_o_)	0.003	0.186	.0370
Gene diversity (*H*_s_)	0.073	0.289	.1430

*Note*: For the presumed apomictic population of *B. laevigata*, only unique MLGs (*n* = 9) were included in the analysis. *R*_S_ is the expected number of alleles per locus at the smallest number of sampled individuals at a locus. *H*_o_ and *H*_s_ (Nei, 1987) are weighted by sample size.

Clonal diversity analyses revealed that although widespread *B. laevigata* has a greater number of observed and expected multilocus genotypes across populations than rare *B. perstellata*, the mean values of diversity indices *D* and *H* were lower for the widespread species than for the rare species (Table 3). Once again, clonal diversity statistics differed greatly among populations of widespread *B. laevigata*. While the TN population of *B. laevigata* exhibited greater *D* and *H* values than any of the populations of rare *B. perstellata*, the IL and PA populations of *B. laevigata* had lower values than any of the *B. perstellata* populations (Table 3). DAPC clustering also revealed greater genetic heterogeneity among populations of widespread *B. laevigata* relative to populations of rare *B. perstellata* (Figure S3). Specifically, all but one of the *B. perstellata* populations clustered together with relatively minor genetic differences, while the three *B. laevigata* populations were more widely spaced in the analysis output. Each population was its own genetic cluster, except that the relatively high diversity TN population of *B. laevigata* contained three genetic clusters and TN1 population of *B. perstellata* contained two clusters.

**TABLE 3:** Clonal diversity statistics using a clonal threshold of 0 for individuals grown in growth chambers from seed collected from four natural populations of rare *Boechera perstellata* and three populations of widespread *B. laevigata*.

Population or species	*N*	Multilocus genotypes	Expected multilocus genotypes	*H*	*D*
KY1	40	6	5.90	1.27	0.65
KY2	41	9	8.63	1.55	0.72
TN1	52	15	12.77	2.22	0.85
TN2	55	11	9.64	1.97	0.84
*B. perstellata*	47	10.3 ± 1.89	9.2 ± 1.41	1.75 ± 0.87	0.765 ± 0.05
IL	60	9	6.74	0.93	0.384
PA	38	1	1.00	0.00	0.000
TN	53	52	37.49	3.94	0.999
*B. laevigata*	50.3	20.7 ± 15.8	15.1 ± 11.3	1.62 ± 1.19	0.461 ± 0.29

*Note*: Statistics include the number of multilocus genotypes, expected number of multilocus genotypes at the least common sample size ≥10 based on rarefaction, Shannon‐Wiener index of multilocus genotype diversity (*H*), and Simpson's index of diversity (*D*). Values shown are means across 17 microsatellite loci ± standard errors (when applicable). Populations of *B. perstellata*: Franklin County, Kentucky, USA (KY1, KY2); Rutherford County, Tennessee, USA (TN1); Smith County, Tennessee (TN2). Populations of *B. laevigata*: Cook County, Illinois, USA (IL); Clarion County, Pennsylvania, USA (PA); Cheatham County, Tennessee, USA (TN).

## 4 DISCUSSION

Phenotypic plasticity can influence organismal fitness and species performance by facilitating expansion across diverse abiotic environments and/or persistence in locations experiencing environmental change (Godoy et al., 2012; Nicotra & Davidson, 2010). For rare endemic species, limited phenotypic plasticity could constrain their geographic range and/or habitat specificity, while widespread species with broader habitat breadths could be facilitated by relatively high phenotypic plasticity (Murray et al., 2002). However, our findings suggest that the ability of phenotypic plasticity to elucidate the relative rarity and commonness of *B. perstellata* and *B. laevigata*, respectively, is limited. Specifically, we found no evidence of plasticity of any measured traits for either species within the context of light and limited differences in plasticity between species within the context of temperature and water. But we suggest that the differences that we did detect in the plasticity of root traits of rare *B. perstellata* and widespread *B. laevigata* in response to those abiotic factors could help to explain differences in their geographic range sizes and habitat associations.

Previous research suggested that temperature plasticity in particular could be especially influential to range size of *Boechera* species (Lovell & McKay, 2015), although other studies of plasticity of rare and common species within the context of temperature did not reveal similar trends (Hirst et al., 2017). Our significant plasticity findings suggest that widespread *B. laevigata* may be able to alter its root allocation in response to warming in ways that *B. perstellata* cannot. The specific responses of *B. laevigata* to warming revealed by our study could indicate increased investment in longer but thinner roots, which could increase the acquisition of belowground resources when they are limited with less total root biomass investment (see Ostonen et al., 2007). Although we did not measure soil moisture of pots in our high‐temperature treatment level, it is plausible that warming would be associated with reduced soil moisture and that plasticity of root traits could facilitate the distribution of *B. laevigata* across a broader latitudinal range than the constrained range of *B. perstellata*. In contrast to their warming responses, root traits of *B. perstellata* and *B. laevigata* both responded to changes in water availability in similar directions. Their shared reductions in root biomass and allocation in response to increased watering could indicate a shift away from strategies that would enhance the acquisition of belowground resources when those resources become more plentiful, which could facilitate their association with habitats ranging from fairly dry to moist. We suggest that the stronger plasticity of root traits in response to water availability of *B. laevigata* could elucidate its association with a wider range of substrates than rare *B. perstellata*. The only trait for which rare *B. perstellata* exhibited more plasticity than *B. laevigata* was SLA in response to increased water availability, but in a direction that contrasts other reported responses of SLA to wetter conditions (see Dwyer et al., 2014; Rosas et al., 2019).

In the face of environmental change, limited plasticity could impede species persistence, especially when migration and/or adaptation are also impeded by low fitness and genetic diversity as is characteristic of many rare species (Boyd, Anderson, et al., 2022). In contrast, phenotypic plasticity could provide a pathway to persistence through change, especially relatively rapid environmental change that may outpace the ability of species to adapt to new conditions (Chevin et al., 2010; Snell‐Rood et al., 2018). Habitat loss due to disturbances such as development and timber harvesting remains an ongoing threat to rare *B. perstellata* given its small number of extant occurrences (USFWS, 2004). A lack of phenotypic plasticity of *B. perstellata* in the context of light (which is shared by widespread *B. laevigata*) could limit its ability to respond to such change. Our findings also suggest that climatic warming could threaten the persistence of *B. perstellata* given its limited plasticity in response to warming (Figure 3). Although there were some observable differences in plasticity among populations of both *Boechera* species (Figures 2 and 3), the insignificance of these differences could reflect overall similarities given their shared evolutionary history or be due to relatively small sample sizes within populations that reduced our statistical power to detect population‐level differences compared with differences at the species level.

Importantly, phenotypic plasticity is not always adaptive (Bonser, 2021; Hendry, 2016; Palacio‐López et al., 2015), but research on the adaptive nature of phenotypic plasticity in plant traits has been limited (Arnold et al., 2019; Wei et al., 2021), including within the context of rarity (Boyd, Anderson, et al., 2022). While our selection analyses revealed that plasticity was not always influential to fitness (i.e., plasticity was most neutral), we did find evidence that plasticity in RSR_mass_ across *Boechera* species within the context of water availability is adaptative. Plasticity in root allocation as a response among plants to changes in the availability of water and other belowground resources (Eriz et al., 2017) is a common example of the optimal partitioning theory (see Bloom et al., 1985). Although there is generally a positive association between vegetative size and reproductive output in plant individuals of the same age and species (Weiner et al., 2009), we concede that the use of total biomass as a fitness proxy in our selection analyses may not have resolved the actual fitness consequences of plasticity. Biomass allocation, in particular, involves trade‐offs in investment toward functions including maintenance, growth, and reproduction (Weiner et al., 2009), and we were unable to resolve the relationship between vegetative and reproductive biomass of *Boechera* species in our experiments due to time and space constraints associated with the growth chambers. We suggest that future longer‐term research experiments that perhaps utilize garden and/or field settings include assessments of more direct and lifetime fitness consequences of phenotypic plasticity (Anderson et al., 2021; Baythavong et al., 2011; Baythavong & Stanton, 2010; Van Buskirk & Steiner, 2009).

In contrast to our plasticity results, our analysis of the species‐level genetic diversity of rare *B. perstellata* and widespread *B. laevigata* largely supported our hypothesis about the comparative genetic diversity of these rare and common species, which aligns with the results of previous comparative syntheses (Boyd, Anderson, et al., 2022; Cole, 2003; Gitzendanner & Soltis, 2000; Leimu & Fischer, 2008). Specifically, there were more than twice as many alleles present across sampled loci for widespread *B. laevigata* compared with rare *B. perstellata* even though fewer *B. laevigata* populations were sampled. However, our hypothesis that the widespread species would have higher levels of genetic diversity was not consistently supported at the population level given that the three sampled *B. laevigata* populations diverged greatly in genetic diversity, probably because they represent a range of reproductive modes. Nearly every individual of the highly heterozygous *B. laevigata* population from Illinois shared an identical heterozygote genotype (i.e., fixed heterozygosity) for each polymorphic locus, resulting in a low number of multilocus genotypes and suggesting that this population is engaging in apomixis rather than sexual reproduction. High levels of heterozygosity tend to be associated with apomixis in *Boechera* species (Beck et al., 2012; Li et al., 2017; Rushworth et al., 2011, 2018) and are thought to be the result of hybridization between genetically distinct lineages (Alexander et al., 2015; Beck et al., 2012; Dobeš et al., 2006; Li et al., 2017; Windham et al., 2015). Although *B. laevigata* is known to reproduce sexually via both outcrossing and selfing (Bloom, 1988), apomixis has been previously reported in this species (Carman et al., 2019). In contrast to apomictic lineages, sexual *Boechera* species are usually highly inbred due to selfing and thus have very low levels of heterozygosity (Dobeš et al., 2006; Rushworth et al., 2011, 2018; Windham et al., 2015). The lack of genetic diversity of the Pennsylvania population of *B. laevigata* suggests that this is likely a sexual but highly inbreeding population that may have gone through one or more severe genetic bottlenecks resulting in the loss of alleles. The relatively high diversity population of *B. laevigata* from Tennessee also appears to be reproducing sexually because it lacks fixed heterozygosity, and while this population appears to be inbreeding (as its observed heterozygosity levels are much lower than what would be expected for an outcrossing population), its relatively high measures of genetic diversity indicate that it has not experienced the degree of allele loss through random genetic drift that the Pennsylvania population appears to have experienced. Regardless of the diversity measure examined, this population of *B. laevigata* had much higher genetic diversity than any population of rare *B. perstellata*. Additionally, the wide range of reproductive modes evidenced for *B. laevigata* could facilitate its ability adapt to different population situations. For example, it has been suggested that asexual reproduction (e.g., apomixis) might aid in range expansion (Hörandl, 2006; Meloni et al., 2013; Windham et al., 2015).

It has been broadly hypothesized that species that can tolerate a wide range of environmental conditions (i.e., species with broad niche breadths) would be associated with relatively wide geographic range sizes and diverse habitat types (Brown, 1984; Cardillo et al., 2019; Gaston, 2000; Slatyer et al., 2013). Previous research on rare species reported a positive association between phenotypic plasticity (within the context of edaphic factors) and niche breadth (Rutherford et al., 2017), and this association has been more broadly demonstrated with congeneric comparisons beyond the context of rarity species (e.g., Bell & Sultan, 1999; Griffith & Sultan, 2005; Sultan, 2001). While the plasticity of rare endemic *B. perstellata* in response to water suggests that its fundamental niche may be broader than its current distribution, which could facilitate its persistence in the face of some types of abiotic disturbance, its limited genetic diversity and limited plasticity in the context of temperature could suggest that it is less able to adapt and/or acclimate to the range of temperatures experienced by widespread *B. laevigata* across its broader range. Although the growth chamber‐based approach of our research allowed for tight controls of environmental conditions across which plasticity could be assessed, future transplant experiments of narrowly distributed rare species such as *B. perstellata* beyond their natural ranges could be used to further assess their fundamental niche size compared with more common congeners in “real world” conditions. Such transplants combined with replacement and/or additive experiments also could elucidate the potential role of local edaphic factors and biotic factors such as competition in the restriction of *B. perstellata* to its narrow range and habitat type, especially given the potential for invasive non‐native and prolific native species to encroach into its habitat in response to disturbance (USFWS, 2004). Although our study was focused on just one pair of congeneric species, our experimental approach combined with “real world” transplants could be applied to other rare species toward elucidating broader understanding of species rarity. Such work would need to consider the protected status and imperiled nature of many rare species and integrate careful plans for both research and protection.

## AUTHOR CONTRIBUTIONS

**Jennifer Nagel Boyd:** Conceptualization (equal); data curation (equal); formal analysis (equal); funding acquisition (lead); writing – original draft (lead); writing – review and editing (lead). **Carol Baskauf:** Conceptualization (equal); data curation (equal); formal analysis (equal); funding acquisition (equal); supervision (lead); writing – original draft (supporting); writing – review and editing (supporting). **Annie Lindsay:** Data curation (equal); writing – review and editing (supporting). **Jill T. Anderson:** Conceptualization (equal); formal analysis (equal); writing – original draft (supporting); writing – review and editing (supporting). **Jessica Brzyski:** Conceptualization (equal); funding acquisition (equal); writing – review and editing (supporting). **Jennifer Cruse‐Sanders:** Conceptualization (equal); funding acquisition (equal); writing – original draft (supporting); writing – review and editing (supporting).

### OPEN RESEARCH BADGES

This article has earned an Open Data badge for making publicly available the digitally‐shareable data necessary to reproduce the reported results. The data is available at https://datadryad.org/stash/share/AlNwOP2bbXWggvCmMrnrCl8R3X6pvYjC‐eWOCNl‐ViM.

## Supporting information


Figure S1

Figure S2

Figure S3

Table S1

Table S2

Table S3

Table S4

Table S5

Table S6

Table S7

## Data Availability

The data that support the findings of this study are available in the Dryad repository: https://datadryad.org/stash/share/AlNwOP2bbXWggvCmMrnrCl8R3X6pvYjC‐eWOCNl‐ViM.
