# Supplementary material for: Phenotypic plasticity and genetic diversity shed light on endemism of rare Boechera perstellata and its potential vulnerability to climate warming
Source: Ecol Evol. 2023 Sep 15;13(9):e10540. doi: 10.1002/ece3.10540 (PMC10502469; doi:10.1002/ece3.10540)
Supplement: Supplementary file 1 — Figure S1 [file ECE3-13-e10540-s008.docx]

Boyd et al. – *Ecology and Evolution* – Figure S1

Figure S1. Survival probability of rare *Boechera perstellata* (closed circles, solid lines) and widespread *B. laevigata* (open circles, dashed lines) grown in ambient conditions of *B. perstellata* habitat and with increased light, temperature, and water availability. Values shown are species-level means ± 1 SE of the mean.


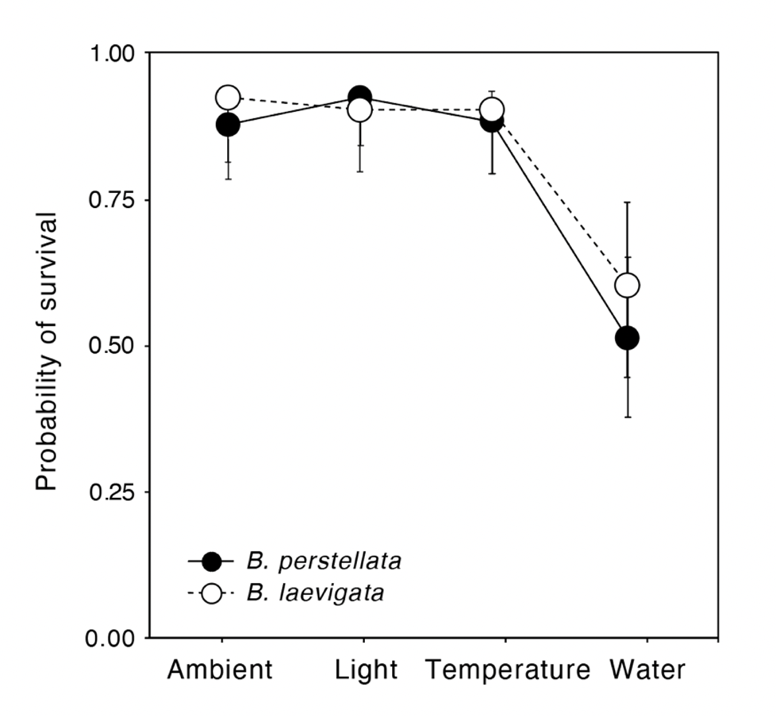


|  | Chisq | df | Pr (>Chisq) |
| --- | --- | --- | --- |
|  |  |  |  |
| Species | 1.0356 | 1 | 0.3088 |
| Treatment | 23.8962 | 3 | ≤ 0.0001 |
| Species × treatment | 1.6832 | 3 | 0.6407 |
| Population | 0.8831 | 1 | 0.3474 |
|  |  |  |  |
