# Supplementary material for: Phenotypic plasticity and genetic diversity shed light on endemism of rare Boechera perstellata and its potential vulnerability to climate warming
Source: Ecol Evol. 2023 Sep 15;13(9):e10540. doi: 10.1002/ece3.10540 (PMC10502469; doi:10.1002/ece3.10540)
Supplement: Supplementary file 2 — Figure S2 [file ECE3-13-e10540-s003.docx]

Boyd et al. – *Ecology and Evolution* – Figure S2

Figure S2. Multivariable genotypic selection analysis of phenotypic plasticity for fitness of individuals of rare *Boechera perstellata* (left side of graphs, closed circles, solid lines) and common *B. laevigata* (right side of graphs, open circles, dashed lines) in comparisons of ambient conditions of *B. perstellata* habitat with increased water conditions. Graphs depict selection on plasticity of the mass-based root-to-shoot ratio (RSR_mass_) of individuals. Fitness was measured as total biomass of individuals. The panels plot partial residuals of genotypic averages from multiple regressions using the predictorEffects function of the R package *effects* (ver. 4.2-0; Fox & Weisberg, 2008).

**LITERATURE CITED**

Fox, J., and S. Weisberg. 2018. Visualizing fit and lack of fit in complex regression models with

predictor effect plots and partial residuals. *Journal of Statistical Software* 87: 1–27.
