# Supplementary material for: Phenotypic plasticity and genetic diversity shed light on endemism of rare Boechera perstellata and its potential vulnerability to climate warming
Source: Ecol Evol. 2023 Sep 15;13(9):e10540. doi: 10.1002/ece3.10540 (PMC10502469; doi:10.1002/ece3.10540)

Boyd et al. – *Ecology and Evolution* – Figure S3

Figure S3. Scatterplot of individuals grown in growth chambers from seed collected from four populations of rare *Boechera perstellata* and three populations of widespread *B. laevigata* on the two principal components of discriminant analysis of principal components (DAPC). There were ten genetic clusters as determined by *K* means clustering, with each population being its own genetic cluster except for the TN population of *B. laevigata*, which consisted of three genetic clusters, and the TN1 population of *B. perstellata*, which consisted of two genetic clusters. A total of 19 principal components and three discriminant functions were retained. Insets show eigenvalues of the analyses.


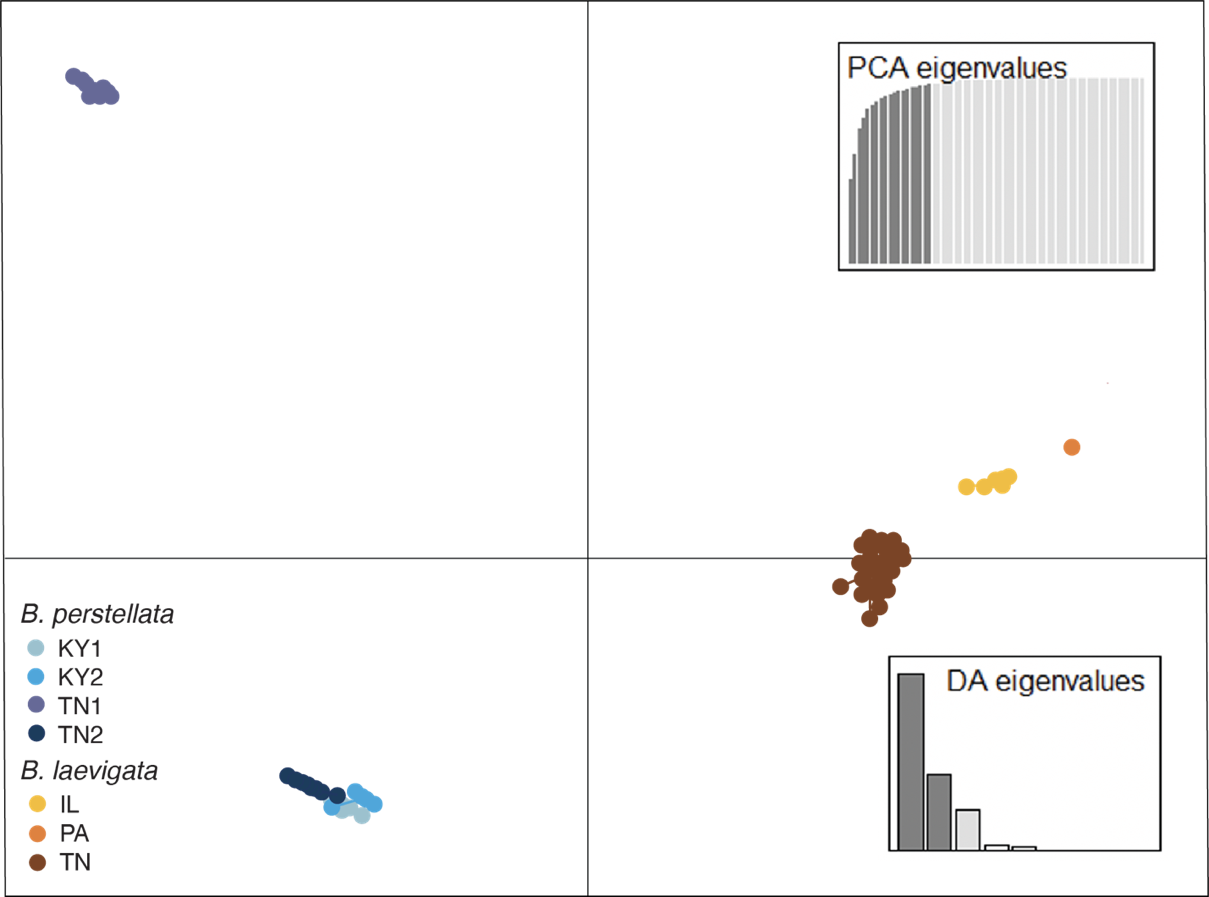

Supplement: Supplementary file 3 — Figure S3 [file ECE3-13-e10540-s007.docx]
