# Supplementary material for: Phenotypic plasticity and genetic diversity shed light on endemism of rare Boechera perstellata and its potential vulnerability to climate warming
Source: Ecol Evol. 2023 Sep 15;13(9):e10540. doi: 10.1002/ece3.10540 (PMC10502469; doi:10.1002/ece3.10540)
Supplement: Supplementary file 4 — Table S1 [file ECE3-13-e10540-s005.docx]

Boyd et al. – *Ecology and Evolution* – Table S1

Table S1. Locations of naturally occurring populations of rare *Boechera perstellata* and widespread *B. laevigata* from which seeds were collected for research. Specific coordinates of *B. perstellata* occurrences are not provided due to the protected status of this species.

| Species | Site | County, State | Latitude | Longitude |
| --- | --- | --- | --- | --- |
|  |  |  |  |  |
| *B. perstellata* | KY1 | Franklin Co., Kentucky | --- | --- |
|  | KY2 | Franklin Co., Kentucky | --- | --- |
|  | TN1 | Rutherford Co., Tennessee | --- | --- |
|  | TN2 | Smith Co., Tennessee | --- | --- |
|  |  |  |  |  |
| *B. laevigata* | IL | Cook Co., Illinois | 42.000568 | -87.464595 |
|  | PA | Clarion Co., Pennsylvania | 41.348516 | -79.220889 |
|  | TN | Cheatham Co., Tennessee | 36.285530 | -87.07982 |
|  |  |  |  |  |
