# Supplementary material for: Phenotypic plasticity and genetic diversity shed light on endemism of rare Boechera perstellata and its potential vulnerability to climate warming
Source: Ecol Evol. 2023 Sep 15;13(9):e10540. doi: 10.1002/ece3.10540 (PMC10502469; doi:10.1002/ece3.10540)
Supplement: Supplementary file 5 — Table S2 [file ECE3-13-e10540-s009.docx]

Boyd et al. – *Ecology and Evolution* – Table S2

Table S2. Numbers of individuals representing four populations of rare *Boechera perstellata* and three populations of widespread *B. laevigata* that survived within abiotic treatment levels.

|  | *B. perstellata* | | |  | *B. laevigata* | | |
| --- | --- | --- | --- | --- | --- | --- | --- |
| Treatment  level | Population | *n* | Survived |  | Population | *n* | Survived |
|  |  |  |  |  |  |  |  |
| Ambient | KY1 | 14 | 12 |  | IL | 16 | 16 |
|  | KY2 | 11 | 10 |  | PA | 12 | 12 |
|  | TN1 | 16 | 15 |  | TN | 16 | 15 |
|  | TN2 | 16 | 16 |  |  |  |  |
|  |  |  |  |  |  |  |  |
| Increased  light | KY1 | 12 | 12 |  | IL | 16 | 16 |
|  | KY2 | 14 | 14 |  | PA | 13 | 12 |
|  | TN1 | 16 | 15 |  | TN | 16 | 15 |
|  | TN2 | 16 | 16 |  |  |  |  |
|  |  |  |  |  |  |  |  |
| Increased  temperature | KY1 | 14 | 12 |  | IL | 16 | 16 |
|  | KY2 | 14 | 13 |  | PA | 12 | 11 |
|  | TN1 | 16 | 15 |  | TN | 16 | 15 |
|  | TN2 | 16 | 16 |  |  |  |  |
|  |  |  |  |  |  |  |  |
| Increased  water | KY1 | 12 | 5 |  | IL | 16 | 13 |
|  | KY2 | 14 | 7 |  | PA | 12 | 5 |
|  | TN1 | 16 | 9 |  | TN | 15 | 8 |
|  | TN2 | 15 | 9 |  |  |  |  |
| Populations of *B. perstellata*: Franklin County, Kentucky, USA (KY1, KY2); Rutherford County, Tennessee, USA (TN1); Smith County, Tennessee (TN2). Populations of *B. laevigata*: Cook County, Illinois, USA (IL); Clarion County, Pennsylvania, USA (PA); Cheatham County, Tennessee, USA (TN). | | | | | | | |
|  | | | | | | | |
