# Supplementary material for: Phenotypic plasticity and genetic diversity shed light on endemism of rare Boechera perstellata and its potential vulnerability to climate warming
Source: Ecol Evol. 2023 Sep 15;13(9):e10540. doi: 10.1002/ece3.10540 (PMC10502469; doi:10.1002/ece3.10540)
Supplement: Supplementary file 6 — Table S3 [file ECE3-13-e10540-s004.docx]

Boyd et al. – *Ecology and Evolution* – Table S3

Table S3. Effects of species and abiotic factors (light, temperature, and water) on phenotypic traits of rare *Boechera perstellata* and widespread *B. laevigata*.

|  |  | Light | |  | Temperature | |  | Water | |
| --- | --- | --- | --- | --- | --- | --- | --- | --- | --- |
| Phenotypic trait | Effect | χ^2^ | p |  | χ^2^ | p |  | χ^2^ | p |
|  |  |  |  |  |  |  |  |  |  |
| Plant height | Species | 1.9053 | 0.5025 |  | 1.4522 | 0.4401 |  | 3.9754 | 0.0890 |
|  | Abiotic factor | 0.9559 | 0.5213 |  | 2.0771 | 0.3105 |  | 4.3469 | 0.0770 |
|  | Species $\times$ abiotic factor | 0.4252 | 0.6037 |  | 0.3185 | 0.6721 |  | 4.7476 | 0.0660 |
|  |  |  |  |  |  |  |  |  |  |
| Number of leaves | Species | 14.3107 | 0.0042* |  | 16.9834 | 0.0003* |  | 17.7011 | 0.0002* |
|  | Abiotic factor | 0.1573 | 0.7183 |  | 0.0004 | 0.9849 |  | 1.0795 | 0.3842 |
|  | Species $\times$ abiotic factor | 0.4850 | 0.5967 |  | 0.6658 | 0.5329 |  | 2.0795 | 0.2240 |
|  |  |  |  |  |  |  |  |  |  |
| Root length | Species | 0.2518 | 0.6928 |  | 0.2375 | 0.7042 |  | 0.1987 | 0.7377 |
|  | Abiotic factor | 1.1808 | 0.5346 |  | 0.0208 | 0.9562 |  | 4.7560 | 0.0717 |
|  | Species $\times$ abiotic factor | 0.8484 | 0.5355 |  | 0.4919 | 0.5929 |  | 2.2596 | 0.2109 |
|  |  |  |  |  |  |  |  |  |  |
| Shoot mass | Species | 1.1467 | 0.5116 |  | 1.2287 | 0.4517 |  | 1.1423 | 0.3850 |
|  | Abiotic factor | 1.4062 | 0.4895 |  | 5.5435 | 0.0626 |  | 1.7876 | 0.2575 |
|  | Species $\times$ abiotic factor | 1.5001 | 0.5417 |  | 0.9131 | 0.4580 |  | 0.6708 | 0.5066 |
|  |  |  |  |  |  |  |  |  |  |
| Root mass | Species | 3.3303 | 0.2295 |  | 4.0278 | 0.1209 |  | 7.5868 | 0.0176* |
|  | Abiotic factor | 0.7630 | 0.5163 |  | 5.3253 | 0.0631 |  | 42.5671 | <0.0001* |
|  | Species $\times$ abiotic factor | 0.1628 | 0.7415 |  | 5.6312 | 0.0680 |  | 7.6744 | 0.0189* |
|  |  |  |  |  |  |  |  |  |  |
| Root:shoot ratio_mass_ | Species | 5.3353 | 0.1411 |  | 19.2560 | 0.0002* |  | 10.6650 | 0.0049* |
|  | Abiotic factor | 0.5273 | 0.6014 |  | 33.1970 | <0.0001* |  | 57.5750 | <0.0001* |
|  | Species $\times$ abiotic factor | 3.7624 | 0.2831 |  | 15.9970 | 0.0004* |  | 14.0190 | 0.0012* |
|  |  |  |  |  |  |  |  |  |  |
| Root:shoot ratio_ength_ | Species | 0.7792 | 0.5363 |  | 1.1345 | 0.4555 |  | 0.3025 | 0.6836 |
|  | Abiotic factor | 1.0089 | 0.5319 |  | 1.1057 | 0.4395 |  | 0.01730 | 0.9297 |
|  | Species $\times$ abiotic factor | 0.0068 | 0.9342 |  | 0.0064 | 0.9723 |  | 3.5924 | 0.1045 |
|  |  |  |  |  |  |  |  |  |  |
| Specific root length | Species | 1.4340 | 0.5200 |  | 0.9604 | 0.4648 |  | 0.1674 | 0.7370 |
|  | Abiotic factor | 3.7459 | 0.2382 |  | 13.4105 | 0.0014* |  | 10.9003 | 0.0052* |
|  | Species $\times$ abiotic factor | 3.5846 | 0.2249 |  | 8.5618 | 0.0154* |  | 0.0112 | 0.9157 |
|  |  |  |  |  |  |  |  |  |  |
| Specific leaf area | Species | 6.5069 | 0.0967 |  | 3.3826 | 0.1483 |  | 5.0339 | 0.0671 |
|  | Abiotic factor | 7.2409 | 0.0962 |  | 3.9216 | 0.1170 |  | 2.4140 | 0.2029 |
|  | Species $\times$ abiotic factor | 1.7377 | 0.5061 |  | 1.2867 | 0.4620 |  | 8.6797 | 0.0124* |
|  |  |  |  |  |  |  |  |  |  |
| Asterisks denote significance at p ≤ 0.05. We show p-values corrected for multiple testing via the Benjamini-Hochberg false discovery rate procedure. Df = 1 for all tests. | | | | | | | | | |
|  |  |  |  |  |  |  |  |  |  |
