# Supplementary material for: Phenotypic plasticity and genetic diversity shed light on endemism of rare Boechera perstellata and its potential vulnerability to climate warming
Source: Ecol Evol. 2023 Sep 15;13(9):e10540. doi: 10.1002/ece3.10540 (PMC10502469; doi:10.1002/ece3.10540)
Supplement: Supplementary file 7 — Table S4 [file ECE3-13-e10540-s001.docx]

Boyd et al. – *Ecology and Evolution* – Table S4

Table S4. Means ± standard errors and numbers of observations (*n*; in parentheses) of measured traits of rare *Boechera perstellata* and widespread *B. laevigata* across ambient and altered abiotic treatment levels in light, temperature, and water experiments.

|  | Light experiment | |  | Temperature experiment | |  | Water experiment | |
| --- | --- | --- | --- | --- | --- | --- | --- | --- |
| Phenotypic trait | *B. perstellata* | *B. laevigata* |  | *B. perstellata* | *B. laevigata* |  | *B. perstellata* | *B. laevigata* |
|  |  |  |  |  |  |  |  |  |
| Plant height (cm) | 6.02 ± 0.14 (*109*) | 5.51 ± 0.17 (*85*) |  | 6.56 ± 0.14 (*109*) | 5.87 ± 1.84 (*84*) |  | 5.63 ± 0.20 (*83*) | 5.10 ± 0.21 (69*)* |
|  |  |  |  |  |  |  |  |  |
| Number of leaves | 55.17 ± 1.80 (*109*) | 36.68 ± 1.52 (*85*) |  | 55.28 ± 1.63 (*109*) | 36.64 ± 1.59 (*84*) |  | 53.16 ± 2.19 (*83*) | 36.07 ± 1.70 (*69*) |
|  |  |  |  |  |  |  |  |  |
| Root length (cm) | 14.55 ± 0.22 (*75*) | 14.91 ± 0.61 (*64*) |  | 13.91 ± 0.23 (*75*) | 14.69 ± 0.65 (*61*) |  | 14.64 ± 0.34 (*64*) | 13.72 ± 0.75 (*55*) |
|  |  |  |  |  |  |  |  |  |
| Shoot mass (g) | 0.97 ± 0.05 (*74*) | 0.90 ± 0.04 (*65*) |  | 1.00 ± 0.05 (*75*) | 0.93 ± 0.04 (*64*) |  | 0.99 ± 0.06 (*67*) | 0.87 ± 0.41 (*58*) |
|  |  |  |  |  |  |  |  |  |
| Root mass (g) | 0.90 ± 0.04 (*74*) | 1.03 ± 0.06 (*65*) |  | 0.81 ± 0.04 (*75*) | 0.90 ± 0.05 (*64*) |  | 0.65 ± 0.03 (*67*) | 0.72 ± 0.06 (*58*) |
|  |  |  |  |  |  |  |  |  |
| Root:shoot ratio_mass_ (g/g) | 1.03 ± 0.05 (*74*) | 1.17 ± 0.06 (*65*) |  | 0.87 ± 0.03 (*75*) | 1.01 ± 0.05 (*64*) |  | 0.76 ± 0.04 (*67*) | 0.94 ± 0.08 (*58*) |
|  |  |  |  |  |  |  |  |  |
| Root:shoot ratio_length_ (cm/cm) | 2.63 ± 0.11 (*74*) | 2.84 ± 0.18 (*62*) |  | 2.35 ± 0.11 (*74*) | 2.54 ± 0.15 (*60*) |  | 3.07 ± 0.26 (*64*) | 2.87 ± 0.19 (*54*) |
|  |  |  |  |  |  |  |  |  |
| Specific root length (cm/g) | 2.83 ± 0.16 (*73*) | 2.79 ± 0.23 (*63*) |  | 2.88 ± 0.14 (*74*) | 2.93 ± 0.23 (*62*) |  | 5.10 ± 0.61 (*63*) | 5.43 ± 0.90 (*55*) |
|  |  |  |  |  |  |  |  |  |
| Specific leaf area (cm^2^/g) | 131.16 ± 4.11 (*98*) | 101.59 ± 3.47 (*84*) |  | 133.06 ± 4.22 (*101*) | 105.92 ± 3.49 (*85*) |  | 142.15 ± 4.71 (*76*) | 116.90 ± 3.97 (*69*) |
|  |  |  |  |  |  |  |  |  |
|  |  |  |  |  |  |  |  |  |
| Significance of species main effects is provided in Appendix S4. | | | | | | | | |
