# Supplementary material for: Phenotypic plasticity and genetic diversity shed light on endemism of rare Boechera perstellata and its potential vulnerability to climate warming
Source: Ecol Evol. 2023 Sep 15;13(9):e10540. doi: 10.1002/ece3.10540 (PMC10502469; doi:10.1002/ece3.10540)
Supplement: Supplementary file 8 — Table S5 [file ECE3-13-e10540-s010.docx]

Boyd et al. – *Ecology and Evolution* – Table S5

Table S5. Multivariable genotypic selection analysis across temperature environments with fitness (total biomass) analyzed as a function of mean trait values, plasticity (RDPI, relative distances plasticity index), and species for traits in which there was significant evidence for plasticity.

|  | Chisq | df | Pr (>Chisq) |
| --- | --- | --- | --- |
|  |  |  |  |
| Mean root:shoot ratio_mass_ | 4.0722 | 1 | 0.0436 |
| Mean specific root length | 0.1342 | 1 | 0.7141 |
| RDPI root:shoot ratio_mass_ | 0.0956 | 1 | 0.7572 |
| RDPI specific root length | 1.8415 | 1 | 0.1748 |
| Species × root:shoot ratio_mass_ | 13.0604 | 1 | 0.0003* |
| Species × specific root length | 2.1449 | 1 | 0.1430 |
| Species × RDPI root:shoot ratio_mass_ | 0.0342 | 1 | 0.8571 |
| Species × RDPI specific root length | 4.265 | 1 | 0.0389 |
|  |  |  |  |
| Asterisks denote significance at Bonferroni-corrected α = 0.025 (= 0.05/2). | | | |
