# Supplementary material for: Phenotypic plasticity and genetic diversity shed light on endemism of rare Boechera perstellata and its potential vulnerability to climate warming
Source: Ecol Evol. 2023 Sep 15;13(9):e10540. doi: 10.1002/ece3.10540 (PMC10502469; doi:10.1002/ece3.10540)
Supplement: Supplementary file 9 — Table S6 [file ECE3-13-e10540-s006.docx]

Boyd et al. – *Ecology and Evolution* – Table S6

Table S6. Multivariable genotypic selection analysis across water environments with fitness (total biomass) analyzed as a function of mean trait values, plasticity (RDPI, relative distances plasticity index), and species for traits in which there was significant evidence for plasticity.

|  | Chisq | df | Pr (>Chisq) |
| --- | --- | --- | --- |
|  |  |  |  |
| Mean root mass | 181.7792 | 1 | <0.0001* |
| Mean root:shoot ratio_mass_ | 47.3940 | 1 | <0.0001* |
| Mean specific root length | 0.1029 | 1 | 0.7484 |
| Mean specific leaf area | 0.0843 | 1 | 0.7715 |
| RDPI root mass | 3.8503 | 1 | 0.0497 |
| RDPI root:shoot ratio_mass_ | 10.0736 | 1 | 0.0015* |
| RDPI specific root length | 1.4834 | 1 | 0.2232 |
| RDPI specific leaf area | 0.0332 | 1 | 0.8554 |
| Species × root mass | 6.5261 | 1 | 0.0106* |
| Species × root:shoot ratio_mass_ | 1.4312 | 1 | 0.2316 |
| Species × specific root length | 1.3183 | 1 | 0.2509 |
| Species × specific leaf area | 0.0003 | 1 | 0.9864 |
| Species × RDPI root mass | 2.5233 | 1 | 0.1122 |
| Species × RDPI root:shoot ratio_mass_ | 0.0053 | 1 | 0.9417 |
| Species × RDPI specific root length | 0.0051 | 1 | 0.9432 |
| Species × RDPI specific leaf area | 0.7250 | 1 | 0.3945 |
|  |  |  |  |
| Asterisks denote significance at Bonferroni-corrected α = 0.025 (= 0.05/2). | | | |
