# Supplementary material for: Phenotypic plasticity and genetic diversity shed light on endemism of rare Boechera perstellata and its potential vulnerability to climate warming
Source: Ecol Evol. 2023 Sep 15;13(9):e10540. doi: 10.1002/ece3.10540 (PMC10502469; doi:10.1002/ece3.10540)
Supplement: Supplementary file 10 — Table S7 [file ECE3-13-e10540-s002.docx]

Boyd et al. – *Ecology and Evolution* – Table S7

Table S7. Primer pairs used in the genetic analysis of rare *Boechera perstellata* and widespread *B. laevigata*. Source of information is from Schranz et al. (2007) supplementary Table 2 except when footnotes indicate otherwise.

| Locus | Primer sequence  (F = forward, R = reverse) | Repeat motif and  number from source |
| --- | --- | --- |
|  |  |  |
| B07 | F-CGGGAAGATTCAGCAGGTAA  R-TCCTTTCCTCTCTTTATCCATCA | (TTG)_14_ |
| b6^a^ | F-GCAAAAGATCTTCATGGGAC  R-TGCCATTTCTTTCCCTAGTG | CT, GT |
| B11 | F-CCAAAGCAGTGACCAAAACA  R-GAGCAGCATCAGGAGAAACC | (CT)_13_ |
| C02 | F-CTCGGTCTCCTCCATTACCA  R-CGTTGTTTGGTGTCTGCATC | (GA)_16_ |
| C03 | F-CTCGGTCTCCTCCATTACCA  R-CGTTGTTTGGTGTCTGCATC | (GA)_16_ |
| E09 | F-GCGTATCTCGAATCACCTTTG  R-CTCCCCCTGAGTTTTTCAAG | (AAG)_9_ |
| E11 | F-CATTTGGCTGTCCATGTTGA  R-AGGGGTACAAGTGGTGGTTG | (CT)_16_ |
| F03 | F-TCCGCAAAACTAAAAGGCTTA  R-CCATCTTCACTTCCCGATGA | (TC)_14_ |
| G03 | F-CGCCTCCATTTTATCTTCCA  R-GTTGGTAACGCCGAATCTGT | (GA)_16_ |
| G06 | F-TGTGCAGTTAAAGCCATCCA  R-GCCCCCAAATCAACCTCTAT | (CT)_14_ |
| G08 | F-CAGGAGCTGAATGAACTTTGG  R-TGAGCCAGCAGAGCTTAACA | (AG)_20_ |
| G09 | F-CCCCATAGCTTTTTCTTCCA  R-CCAGTCGTGATGTGTTTTAGAGA | (AT)_13_ |
| H06 | F-TGCATTTCACCGTTTCATTT  R-TAATTTTTCCCCGCTCATTT | (AT)_16_ |
| ICE4^a, b^ | F-CACGAGGAATCTGGCATGGTCG  R-AGCGATTGCAAGCGGCTCAAG | CT |
| ICE5^b^ | F-CTTGCAACCGCCAACTCAATCG  R-CCTGTCTCGCTCCCGCACG | GT |
| R3_02 | F-TTAGTGCTCCAAACCCTTCG  R-TTCCAGGCGAGTGAGAAGAT | (CT)_22_ |
| R3_35 | F-TCATCGCCTGCAAGTAACAA  R-CCAGAGGATCTTATCGGTGTAA | (AG)_13_ |
| ^a^ Dobeš et al. 2004  ^b^ Clauss et al. 2002 | | |
|  |  |  |

**LITERATURE CITED**

Clauss, M. J., H. Cobban, and T. Mitchell-Olds. 2002. Cross-species microsatellite markers for

elucidating population genetic structure in *Arabidopsis* and *Arabis* (Brassicaceae). *Molecular Ecology* 11: 591–601.

Dobeš, C., T. Mitchell-Olds, and M. A. Koch. 2004. Intraspecific diveresification in North

American *Boechera stricta* (=*Arabis drummondii*), *Boechera* $\times$ *divericarpa*, and *Boechera holboellii* (Brassicaceae) inferred from nuclear and chroloplast molecular markers – an integrative approach*. American Jounral of Botany* 91: 2087–2101.

Schranz, M.E., A. J. Windsor, B. H. Song, A. Lawton-Rauh, and T. Mitchell-Olds. 2007.

Comparative genetic mapping in *Boechera stricta*, a close relative of Arabidopsis. *Plant*

*Physiology* 144: 586-598.
